# Potentials of Host-Directed Therapies in Tuberculosis Management

**DOI:** 10.3390/jcm8081166

**Published:** 2019-08-03

**Authors:** Yash Dara, Doron Volcani, Kush Shah, Kevin Shin, Vishwanath Venketaraman

**Affiliations:** Department of Basic Medical Sciences, College of Osteopathic Medicine of the Pacific, Western University of Health Sciences, Pomona, CA 91766, USA

**Keywords:** *Mycobacterium tuberculosis*, host-directed therapies, autophagy, immune responses

## Abstract

Tuberculosis (TB) remains as a leading cause of mortality in developing countries, persisting as a major threat to the global public health. Current treatment involving a long antibiotic regimen brings concern to the topic of patient compliance, contributing to the emergence of drug resistant TB. The current review will provide an updated outlook on novel anti-TB therapies that can be given as adjunctive agents to current anti-TB treatments, with a particular focus on modulating the host immune response to effectively target all forms of TB. Additional potential therapeutic pathway targets, including lipid metabolism alteration and vascular endothelial growth factor (VEGF)-directed therapies, are discussed.

## 1. Introduction

Tuberculosis (TB), caused by the bacterial pathogen *Mycobacterium tuberculosis* (*M. tb*), remains a leading cause of mortality in developing countries, with approximately one-third of the global population remaining infected and 10 million new cases reported officially to the World Health Organization (WHO) in 2017 [1]. Currently, the best first-line curative therapy strategy for TB is the Directly Observed Treatment, Short Course (DOTS), comprising of an antibiotic regimen of isoniazid, rifampicin, pyrazinamide, and ethambutol for six to nine months, and additional patient monitoring during the first two initial months. However, due to its long duration and adverse side effects, patient compliance greatly decreases, leading to discontinuation of DOTS. This has contributed to the emergence of multidrug-resistant TB (MDR-TB), representing the continuous threat it possesses to public health and economic growth, globally [2]. As antimicrobial resistance against *M. tb* increases, it has become an international priority to develop effective, novel therapeutic approaches.

Recent improvements in the field of genomics have provided insight into various pathways that can be exploited as targets of possible emerging therapies. Along with the relatively successful treatment methodology of the pre-chemotherapy era, which demonstrated a capacity of ‘self-cure’ against TB, a shift in focus towards modulating the host immune response as adjunctive therapy, namely host-directed therapies (HDTs), to meet the needs for nearly all forms of *M. tb* infections, including MDR-TB, is underway. The emerging and existing HDT agents and their potentials will be the subject of the current review.

## 2. mTOR Inhibition

In recent years, there is an interest in exploiting autophagy pathways via HDTs as an approach to manage TB [3,4]. This cellular process, involving lysosomal degradation, is essential in the removal of protein aggregates and damaged cellular organelles that may damage the integrity of the cell [3]. A summary of the autophagy pathway is described in the following: as autophagy begins, a double-membrane bound vesicle containing cytoplasmic material, namely an autophagosome, is formed. These autophagosomes will fuse with lysosomes to form autophagolyosomes, resulting in degradation of cellular debris [4]. While this process is complex, there are three required components, Unc-51-like Kinase 1 complex (ULK1), focal adhesion kinase family interacting protein of 200 kd (FIP200), and autophagy related protein 13 (ATG13) [4].

Mammalian target of rapamycin (mTOR) is a multi-process regulator, specifically involved in numerous anabolic pathways, thus being most active when nutrients are readily available, aiding in cell survivability [4,5]. Typically, mTOR is inactivated in conditions of cellular stress (i.e., hypoxia and starvation), inducing autophagy. One proposed mechanism of mTOR interaction in autophagy via phosphorylation of ATG13 to inhibit the actions of the ULK complex [5]. Additionally, another discussed mechanism is the inhibition of the active form of Beclin-1-regulated autophagy protein [5]. Downstream metabolic effects after mTOR activation have been previously described: mTOR-1 complex activates SREBP-1 and PPAR-gamma to promote lipogenesis and activates S6K1 to promote glucose transporter 1 (GLUT-1) synthesis to increase glycolysis and lipogenesis. Additionally, mTOR-1 complex activates S6K1 to promote protein synthesis [5].

In the case of TB, the key role of autophagy is to restrict *M. tb* growth and can be triggered to do so via numerous intracellular cues (i.e., IFN-gamma and vitamin D) [5]. However, it has been observed that in the presence of *M. tb*, mTOR activity is continuously expressed, and thus, will promote the aerobic glycolytic pathway [5]. This shift in metabolism, being similar to the Warburg effect in cancer cells, is thought to be essential in mounting an immune response against *M. tb* [6]. Because mTOR is a negative regulator of autophagy, it can be noted that *M. tb* survivability is correlated with the inhibition of autophagolysosome fusion [6]. As such, stimulating autophagy via various pathways will increase the ability to fight *M. tb* infection [5,6,7]. One of the main strategies to tackle *M. tb* is through direct inhibition of the mTOR via rapamycin analogs, with greater focus on everolimus due to its greater bioavailability and lower side effect profile [7]. While the beneficial effects of everolimus has been demonstrated over the years, it is important to note high doses of everolimus is also an effective immunosuppressant and has applications in cases of organ transplants and cancer therapies. There have been discussions of utilizing various drug delivery systems to target *M. tb* specific cell [4]. 

Looking at in vivo effects of rapamycin and everolimus reiterates the potential yet further investigation needed when using mTOR inhibition for treating *M. tb* infection. In a Zebrafish model with *M. marinum* infection, mTOR-deficient Zebrafish cleared infection earlier [8]. In mice models, rapamycin given to mice at an early age did not significantly change life expectancy or susceptibility to disease; however, when given at a later age, the mice had better survival expectancy [9]. When rapamycin was given to BCG-vaccinated mice, there was an increase in the vaccination efficacy against *M. tb* infection associated with autophagy, increased antigen presentation, and increased Th1-type immune response [10]. However, when investigating rapamycin efficiency in cells pre-infected with HIV, the mTOR inhibition was advantageous for *M. tb* [11]. The rapamycin derivative, everolimus, also shows promise in vivo. In healthy elderly volunteers given low doses of everolimus, there was a 20% improvement in protection after the influenza vaccination, with low doses showing the lowest number of adverse events [12]. In contrast, when looking at organ-transplant patients given everolimus, there was a higher risk of TB and reactivation of latent TB [13]. The doses in this study were higher than that of the influenza study, and the higher risk associated with higher doses of everolimus could be overcome with Rifampicin [14]. Overall, more needs to be done to understand the dose-response of everolimus. Alternatively, enhancing the delivery of such drugs could have better potential against *M. tb*. For example, an in vitro study looking at inhalable rapamycin in particles showed “macrophages exposed to the particles or rapamycin in solution at a concentration of 100 μg/mL over a 24 h period maintained 79.37 ± 0.72% and 58.33 ± 1.39% viability, respectively” [15]. They further concluded inhalable rapamycin to be better at clearing *M. tb* than rapamycin in solution in vitro [15].

## 3. Cathelicidin (LL37) Inducers

Cathelicidins are part of the antimicrobial peptide class, with LL37 being the only known human cathelicidin [16]. They kill mycobacteria, regulate the innate immune system through cytokines and chemokines, and participate in autophagy [17]. LL37 is 37 amino acids long, alpha-helical, and cationic making it have a higher affinity to DNA, and aggregates in solution [16]. It penetrates the bacterial membrane, causing pores and bacterial lysis, but it does not affect mammalian cells with cholesterol in their membranes [16]. LL37 is described to have both proinflammatory and anti-inflammatory effects depending on its environment [16]. Cathelicidins are expressed in neutrophils, monocytes, keratinocytes, lymphocytes, and epithelial cells of the skin, testis, gastrointestinal system, and respiratory tract [17].

LL37 produced by alveolar macrophages and neutrophils significantly contributes to the growth inhibition of intracellular pathogens [17]. Specifically, *M. tb* is shown to induce synthesis of LL37 in epithelial cells, neutrophils, monocyte-derived macrophages, and in alveolar macrophages [17]. However, LL37 levels were undetectable in TB granulomas, indicative of its absence during late stages of *M. tb* infection. *M. tb* increases the amount of LL37 by activating Toll-Like Receptors (TLRs) 2, and especially 9 [18]. In this proinflammatory pathway, the activated TLR9 will induce the synthesis of Type I IFN, LL37 and thereby forming M1 macrophages, NETosis (neutrophil extracellular traps) with DNA complexes, and increased inflammatory cells [16]. When LL37 produced during *M*. *tb* infection triggers formation of NETs, the NET: LL37complexes containing *M. tb* will then be internalized by the macrophages, followed by the killing of the pathogen inside the lysosomes [19].

The importance of cathelicidins and their protection against TB are seen in the animal studies below [20,21]. It has been shown that mycobacterial infection can increase the levels of cAMP, which inhibits cathelicidins and therefore promotes intracellular *M. tb* growth. This was demonstrated in mice lacking the cathelicidin-producing gene. This study used Cramp-knocked out mice (the gene equivalent to human LL37 gene), to see if it was required for regulating protective immunity against *M. tb* in vivo. They describe the experiment as using “Cramp−/− mice in a validated model of pulmonary tuberculosis and conducted cell-based assays with macrophages from these mice” [20]. The results showed “macrophages from Cramp−/− mice were unable to control M. tuberculosis growth in an in vitro infection model, were deficient in intracellular calcium influx and were defective in stimulating T-cells [20]. Additionally, CD4 and CD8 T-cells from Cramp−/− mice produced less IFNβ upon stimulation. Furthermore, bacterial-derived cyclic-AMP modulated cathelicidin expression in macrophages” [20]. Thus, it is necessary to have cathelicidins for the innate response to protect against *M. tb* infections [20]. In another study, treatment of immunocompromised mice that are latently infected with *M. tb* with combination of TNF-alpha, beta-defensin, and LL37 resulted in protection and prevented reactivation of latent *M. tb* infection. This further confirms the ability of LL37 in protecting against reactivation of latent *M. tb* infection. Still, there is some caution needed when considering LL37 as a therapeutic, as it has been shown that in people who were resistance to Colistin (an antibiotic usually given as a last line of defense to drug resistant bacteria), were also resistant to LL37 [21].

Recent studies have demonstrated the potential of vitamin D administration to induce LL37 production via activation of TLR2/1 on human macrophages [22,23]. Vitamin D causes increase in cathelicidin release, which upregulates transcription of Beclin-1 and Atg5, which help in autophagy [24]. It is important to note that without LL37, vitamin D does not decrease *M. tb* growth, as demonstrated by using siRNA to knockout LL37 gene [25]. In addition, Vitamin D may also inhibit *M. tb* growth by enhancing the ability of monocytes to respond to IFN-gamma [26]. It then seems promising to use Vitamin D supplementation for individuals more susceptible to *M. tb* infection, such as HIV patients [27]. However, there are conflicting reports regarding the effectiveness of serum-25 hydroxyvitamin D in cases of active pulmonary tuberculosis. In one cross-sectional study, it was determined that HIV patients, testing positive for pulmonary TB, still had greater serum levels of 25-hydroxyvitamin D [27]. While this is at variance with other reports, there are still clinical trials demonstrating beneficial potential of adjunctive vitamin D supplementation. Since vitamin D, as well as 4-phenylbutyrate (PBA), have been shown to induce LL37 release, one study found giving either vitamin D, PBA, or both in conjunction with chemotherapy to adults with active TB provided beneficial effects towards clinical recovery [28]. Currently, there is an ongoing clinical trial looking at the effectiveness of vitamin D supplementation in adjunct to antiretroviral therapy for HIV-infected adults with low serum-25 hydroxyvitamin D since these individuals are at higher risk for mortality, HIV progress, and incidence of TB [29]. This study’s findings are expected to lead to larger vitamin D supplementation trials for preventing pulmonary TB in other populations as well [29].

## 4. Adjunctive Defensin Therapy

Defensins are another group of antimicrobial peptides that are part of the innate immune system [30]. They are arginine rich, cationic, 16–50 amino acids long, that form beta-sheets held by three disulfide bonds. Defensins have pharmaceutical potential due to their low molecular weight, non-specific and broad activity, and are resistant to proteolysis. There are three subfamilies: alpha, beta, and theta. They differ based on their length, disulfide bond locations, and structure of their precursor. Alpha and beta are more similar as compared to theta in amino acid sequence, tertiary structures, and location of their genes on the same chromosome [30]. Defensins are stored in the granules and lysosomes of innate immune and are released into the environment. They help against many pathogens, including gram positive and negative bacteria, mycobacteria, fungi, and viruses [30]. 

There are six subtypes of human alpha defensins. They are stored in neutrophils, as well as tracheal epithelial cells, saliva, mucosa of the cheeks, submandibular glands, and the Paneth cells of the small intestines [30]. Alpha defensins work against gram positive and negative bacteria, mycobacteria, fungi, and viruses. There are 11 subtypes of human beta defensins, which are seen in the intestines, reproductive system, epithelial cells of the trachea and bronchi, mucous, and macrophages [30]. They have antimicrobial effects on gram positive and negative bacteria, multi-resistant bacteria, and yeast. As for the theta defensins, they have not been found in humans, and while they have shown antimicrobial activity, they have not been shown to be effective against mycobacteria [30].

Besides their antimicrobial effects, defensins also increase histamine release by activating mast cells, increase cytokines, act as ligands, help in wound healing, and beta defensins increase bone growth [30].

More specifically, *M. tb* infection is frequently associated with the production of human beta defensin-2 (HBD-2) [31]. It has been shown that *M. tb* induces HBD-2 mRNA expression in lung epithelial cells, and alveolar macrophages. HBD-2 peptides are seen in lung epithelial cells and are more concentrated where there are *M. tb* clusters [31]. During progressive pulmonary TB, there is an initial rise in HBD-2, which inhibits bacilli proliferation. During latent TB, there is a continuous increase in HBD-2, however when TB is reactivated, there is low amounts of defensin [18]. There are also correlations with alpha defensins and TB, as seen when comparing patients with TB and healthy individuals who previously had TB both showing an increase in alpha 1, 3, and 4 defensins, seen in eosinophils [17].

More specific to the subtypes of beta defensins, HBD-1 is constitutively released, whereas 2-4 are induced by pathogens. HBD-1 and 2 are important for permeabilizing *M. tb*., and HBD-2 is especially seen in the respiratory tract where they are induced by bacteria and cytokines, go to macrophages or phagosomes, and are activated when LPS reacts with TLR4 [17,32]. HBD-4 has shown to be released in infected macrophages by TLR2/1 stimulation induced by IL-1B and vitamin D [17,32]. L-isoleucine has also shown to upregulate gene expression of HBD-3 and 4, which lowers the bacilli load [32].

One mechanism as to how defensins work was shown *in vitro*, where they used 50 mg/mL to inhibit *M. tb* growth, independent of calcium and magnesium [33]. Lesions were seen on the surface of the bacteria [33]. This means there is an increase in permeabilizing the cell membranes of the bacteria, which was further shown to occur extracellularly or during phagocytosis. Defensins are also attracted to glycoproteins to kill viruses with envelopes, and polyanionic structures such as DNA. The cationic properties of defensins also enable them to be attracted towards the anionic phospholipids of bacteria and viruses, in addition to DNA [32].

Another mechanism of defensins is their chemotactic effects [32]. Alpha defensins recruit macrophages, while beta defensins recruit immature T-cells and dendritic cells through CCR6 receptors. Theta defensins have no chemotactic effects [32]. *M. tb* causes macrophages to release HBD-2 through TLR and recognizing pathogen-associated molecular patterns such as peptidoglycan and lipoteichoic acid. This is done through MAPK or NF-kB, which further causes proinflammation and cytokines to be released [32].

Looking from a future pharmaceutical and therapeutic perspective, some of the challenges include the excessive cost, increase in concentration causing cytotoxicity, and being less stable in vivo [30,32]. Still, there have been positive signs in using defensins at much lower concentrations in vivo. In this study, mice infected with *M. tb* needed 5 μg of HNP-1 given subcutaneously to have a significant decrease in CFU from the lungs in a matter of 1 week. Even further, “therapy with 1 μg of HNP-1 also resulted in a significant decrease in CFU from lungs after 2 and 4 weeks (*P* < 0.01 for both time points) compared to controls” [34]. Alternatively, another in vivo study successfully used L-isoleucine to induce beta-defensins. Mice were infected with *M. tb* for 60 days were then given 250 µg of intratracheal L-isoleucine every 48 h. The results showed “administration of l-isoleucine induced a significant increase of beta-defensins 3 and 4 which was associated with decreased bacillary loads and tissue damage” [35]. However, another challenge to be aware of is how bacteria resistant to antibiotics with the *mprF* gene seem also to be resistant to antimicrobial peptides. This is because that gene makes the pathogen less negatively charged. However, because defensins are non-specific, there is overall fewer resistant bacteria to this anti-microbial peptide (AMP) compared to antibiotics [32]. There have been few reports of administering defensins as therapy. Most promising is their synergistic effects with anti-Tb drugs, by increasing the permeability of the bacteria, allowing the drugs to interact with its intracellular target more easily [32]. More research should investigate the effects of L-isoleucine and vitamin D as well.

## 5. Metformin

Metformin, a widely known diabetes drug, is one of the front-line medications in the management of type 2 diabetes. Metformin promotes autophagy by altering the AKT-mTOR signaling pathway [36]. It increases phosphorylation of AMPK and decreases the production of various cytokines [36]. Experimental TB mice model studies demonstrated the potential benefits metformin has in improving pulmonary pathology and reducing bacterial load. However, modest effects were shown when metformin was used as an adjunctive therapy with isoniazid, a common anti-TB drug [37]. With recent cohort studies performed in India and Taiwan, there is increasing evidence suggesting metformin usage being associated with a lower risk of incident TB [38,39]. Further clinical research is needed in thoroughly understanding the role of metformin in diabetic patients infected with TB.

## 6. Statins

Altering lipid metabolism via statin therapy is another potential target for novel TB management therapies [7]. Statins (HMG-CoA reductase inhibitors) are utilized to lower serum LDL cholesterol to prevent issues like atherosclerosis. In addition, statins are also known to have anti-inflammatory effects mediated by transforming growth factor-beta and peroxisome proliferator-activated receptor-gamma [40]. In the case of TB, statins promote phagosome maturation and induce autophagy through reductions in cholesterol levels since mycobacteria preferentially utilizes lipid carbon sources as nutrients [41]. Experimental studies using the statin simvastatin as an adjunctive therapy in conjunction with rifampicin, isoniazid, and pyrazinamide resulted in significant reduction in the bacterial burden [42]. Despite being very promising, a large retrospective analysis of statin usage for diabetic patients infected with *M. tb* were not associated with a protective effect [43]. Therefore, it is essential to perform additional research in this area to determine optimal statin agents, including repurposing current FDA-approved drugs, with appropriate dosing schedules for the most effective results. 

## 7. Additional Approaches

Additional emerging approaches that have not been discussed extensively in the literature include nanoparticles, vitamin A supplementation, and anti-VEGF inhibitors. 

Nanoparticles show high promise as an innovative drug delivery platform by providing a controlled environment in which various drugs can be continuously released to improve treatment outcomes [44]. Numerous studies have demonstrated enhanced intracellular accumulation of three anti-TB drugs, namely isoniazid, rifampin, and streptomycin [44]. Antimicrobial activity of isoniazid and streptomycin was enhanced, while there was no notable change in rifampin activity while encapsulated in nanoparticle [44]. As improvements in stability of these nanoparticles system progress, it is highly possible that novel adjunctive therapies can be administered via nanoparticle encapsulation [44]. The other potential with using nanoparticles is lowering cytotoxicity, as demonstrated in a Zebrafish model of tuberculosis study, when thioridazine was encapsulated in a nanoparticle, no toxicity was detected compared to free thioridazine. This study also found the thioridazine nanoparticle therapy improved rifampicin’s effect against *M. tb* in vivo [45]. 

Vitamin A deficiency has been observed in patients with TB. The deficiency of vitamin A in patients with TB might be a contributor to the development of TB [46]. Alternatively, deficiency could be the result of loss of appetite, poor intestinal absorption, increased urinary loss of vitamin A due to TB [46]. Vitamin A deficiency lowers immunity whilst vitamin A supplementation reduces morbidity and mortality, particularly from measles and diarrhea. Vitamin A supplementation also decreases the mortality rate in HIV-infected children and delays the progression to AIDS in HIV infected subjects [46]. A higher incidence of lung cancer and increased mortality have been observed in smokers after beta-carotene supplementation. It is thought that multiple micronutrients rather than vitamin A alone may be more beneficial in patients with TB. However, further research is needed to be conclusive. 

Since TB granulomas feature abnormal vasculature, there is discussion of administering anti-TB drugs in conjunction with antiangiogenic (anti-VEGF) agents, such as bevacizumab [47]. This has the potential to improve delivery of antimicrobial drugs into granulomas and stimulate sensitivity to drug treatment through improved oxygen delivery into the lesions during the window of normalization [47]. Furthermore, this type of ‘host-directed therapy’ that targets the abnormal granuloma vasculature and reduces hypoxia may lead to a more robust immune response again the bacteria. Through this mechanism, vessel normalization has the potential to reduce the overall duration of TB chemotherapies and possibly avoid localized exposure of the bacterium to monotherapy, thereby avoiding the development of drug resistance, which is one of the main issues in the global control of TB [47]. 

## 8. Conclusions

A current review of the developing HDT agents presents as a challenge due to the broad spectrum of research within the field. Current data, both pre- and clinical, is insufficient to draw major conclusions regarding the efficacy and potency of these developing therapy strategies (summary of recent studies has been summarized in Table 1). In addition, it is important to note that most of these agents still need to be researched in clinical settings. At the time of writing this review article, there are three phase 2 clinical trials, NCT0296892 (everolimus, auranofin, vitamin D, CC-11050), NCT03160638 (azithromycin), and NCT03281226 (N-acetylcysteine), that are active, with two of them recruiting patients to demonstrate the efficacies of these various HDT therapy strategies. These data sets can potentially change current anti-TB protocols and induce a shift in direction for anti-TB therapies.

By modulating key host proteins, there remains the possibility of adverse unintended consequences. Nonetheless, novel approaches using HDTs can also provide with benefits in additional contexts, including immune responses to other organisms that may not pertain to TB infections. Additionally, it is just as important to identify new biomarkers to diagnose TB earlier with the hopes of preventing disease progression. Current TB diagnostic methods present with serious limitations in either precision or efficacy. This delays the diagnosis of TB significantly, further aiding in the complexity of the disease. With current advancements in biosensing technologies and point-of-care diagnostic tools, there is large potential to improve detection of TB. These approaches to managing and diagnosing TB, along with advancements in genomics and technology, can result in precise and effective novel therapies with the hopes of mitigating the global burden mycobacterial infections present. From a clinical perspective, these approaches are still in a relative infancy. 

## Figures and Tables

**Table 1:** Host-Directed Therapies against TB: Recent Clinical and Additional Studies.

Candidate	Description	Results	Remarks	References
**1. Clinical Studies**				
Everolimus, Auranofin, Vitamin D, CC-11050	Combination therapy of HDT with DOTS drug regimen, followed with a modified DOTs protocol for 4 months with the intent to improve efficacy and outcomes of TB	Active, not enrolling.	Randomized, phase 2 clinical trial in South Africa	ClinicalTrials.gov Identifier: NCT02968927 [48,49]
Azithromycin	Immunomodulatory, adjunctive HDT therapy on top of the current DOTs regimen to reduce excessive inflammation, tissue degradation, and improve clinical outcomes of TB	Active, enrolling	Prospective, randomized, phase 2 pilot study in the Netherlands	ClinicalTrials.gov Identifier: NCT03160638
N-acetylcysteine	N-acetylcysteine in conjunction with rifampicin, isoniazid, pyrazinamide, ethambutol to provide anti-TB and antioxidative effects for patients with active HIV/TB infections	Active, enrolling	Randomized, phase 2 clinical trial in Brazil	ClinicalTrials.gov Identifier: NCT03281226
Vitamin D	Adjunctive vitamin D therapy in combination to standard antibiotic treatment for pulmonary tuberculosis to potentially enhance patient response	Vitamin D supplementation did not significantly reduce sputum conversion time among study population	Double-blind, randomized phase 3 clinical trial in the United Kingdom	ClinicalTrials.gov Identifier: NCT00419068 [50]
	Vitamin D supplementation to standard DOTs therapy with the hopes of quicker patient recovery times (demonstrated by sputum culture conversion)	Vitamin D supplementation did not significantly reduce sputum conversion time	Double-blind, randomized, placebo-controlled phase 3 clinical trial in South India	ClinicalTrials.gov Identifier: NCT00366470 [51]
	Effects of adjunctive vitamin D on host immunity with respect to TB and response to appropriate treatment	High-dose vitamin D3 corrected deficiency among patient, but did not improve TB clearance over the course of the trial	Double-blind, randomized, controlled phase 2 clinical trial in the United States	ClinicalTrials.gov Identifier: NCT00918086 [52]
	Determining if replacement of vitamin D in deficient patients with active TB affects clinical outcome	Vitamin D in high doses resulted in improvement in all TB patients, including those with vitamin D deficiencies.	Randomized, placebo-controlled clinical trial in Pakistan	ClinicalTrials.gov Identifier: NCT01130311 [53,54]
	Vitamin D and L-arginine supplementation in diagnosed TB patients in order to improve clinical outcomes and responses to pulmonary TB	With the doses administered, neither vitamin D nor L-arginine supplementation affected TB outcomes	Randomized, double-blind, placebo-controlled phase 3 clinical trial in Indonesia	ClinicalTrials.gov Identifier: NCT00677339 [55]
	Vitamin D3 and phenylbutyrate supplementation to standard short course DOTS therapy in order to improve recovery times and improve clinical outcomes in newly diagnosed TB patients	Beneficial effects towards patient recovery has been observed with phenylbutyrate, vitamin D3, or combination of phenylbutyrate and vitamin D3 supplementation with standard short-course therapy	Randomized, double-blind, placebo-controlled, 4-arm Phase 2 clinical trial in Bangladesh	ClinicalTrials.gov Identifier: NCT01580007 [28]
**2. Additional Studies**				
Metformin	Multiple studies investigating the supplementation of metformin to existing standard anti-tuberculosis therapies, specifically in application to diabetic-TB patients	Metformin as an adjunctive therapy for diabetic TB patients needs to be understood further, even at the clinical level, due to inconsistent outcome reporting across studies	Retrospective cohort or case control studies. While there are reports of positive effects of metformin on active TB infections, there has been reports of no significant benefits in utilizing metformin as an adjunctive therapy.	[38,39,56,57,58,59]
Statins	Studies sought to understand the usage of cholesterol-lowering lipids (i.e., statins) and outcomes in regard to TB infections	Statins show beneficial effects as adjunctive therapy in TB infected *M. marinum* TB, and have been observed to shorten the culture negativity, reduce tissue pathology, and enhance bacterial killing along standard TB therapy. However, statins did not prevent TB progression in individuals who were newly diagnosed with type 2 diabetes mellitus.	Further studies will need to be conducted in order to understand the effects of statins and other potential cholesterol-lowering drugs on recovery times and outcome improvement in TB infections.	[42]
